# Consistent Pulmonary and Systemic Responses from Inhalation of Fine Concentrated Ambient Particles: Roles of Rat Strains Used and Physicochemical Properties

**DOI:** 10.1289/ehp.7868

**Published:** 2005-06-27

**Authors:** Urmila P. Kodavanti, Mette C. Schladweiler, Allen D. Ledbetter, John K. McGee, Leon Walsh, Peter S. Gilmour, Jerry W. Highfill, David Davies, Kent E. Pinkerton, Judy H. Richards, Kay Crissman, Debora Andrews, Daniel L. Costa

**Affiliations:** 1 Pulmonary Toxicology Branch, Experimental Toxicology Division, National Health and Environmental Effects Research Laboratory, Office of Research and Development, U.S. Environmental Protection Agency, Research Triangle Park, North Carolina, USA; 2 Center for Environmental Medicine, Asthma, and Lung Biology, School of Medicine, University of North Carolina at Chapel Hill, Chapel Hill, North Carolina, USA; 3 Center for Health and the Environment, University of California at Davis, Davis, California, USA

**Keywords:** concentrated ambient particles, fibrinogen, γ-glutamyltransferase, hypertensive rats, lung inflammation, macrophages, neutrophils, Wistar Kyoto rats

## Abstract

Several studies have reported health effects of concentrated ambient particles (CAP) in rodents and humans; however, toxicity end points in rodents have provided inconsistent results. In 2000 we conducted six 1-day exposure studies where spontaneously hypertensive (SH) rats were exposed to filtered air or CAPs (≤ 2.5 μm, 1,138–1,765 μg/m^3^) for 4 hr (analyzed 1–3 hr afterward). In seven 2-day exposure studies in 2001, SH and Wistar Kyoto (WKY) rats were exposed to filtered air or CAP (≤ 2.5 μm, 144–2,758 μg/m^3^) for 4 hr/day × 2 days (analyzed 1 day afterward). Despite consistent and high CAP concentrations in the 1-day exposure studies, no biologic effects were noted. The exposure concentrations varied among the seven 2-day exposure studies. Except in the first study when CAP concentration was highest, lavageable total cells and macrophages decreased and neutrophils increased in WKY rats. SH rats demonstrated a consistent increase of lavage fluid γ -glutamyltransferase activity and plasma fibrinogen. Inspiratory and expiratory times increased in SH but not in WKY rats. Significant correlations were found between CAP mass (microgram per cubic meter) and sulfate, organic carbon, or zinc. No biologic effects correlated with CAP mass. Despite low chamber mass in the last six of seven 2-day exposure studies, the levels of zinc, copper, and aluminum were enriched severalfold, and organic carbon was increased to some extent when expressed per milligram of CAP. Biologic effects were evident in those six studies. These studies demonstrate a pattern of rat strain–specific pulmonary and systemic effects that are not linked to high mass but appear to be dependent on CAP chemical composition.

A number of studies have recently reported pulmonary and cardiovascular health effects of concentrated ambient particles (CAP) in animals and humans (Clarke et al. 1999, 2000; Ghio et al. 2000; Gordon et al. 2000; Huang et al. 2003; Kodavanti et al. 2000a; Saldiva et al. 2002; Smith et al. 2003). Real-time ambient particle concentrators are designed to concentrate ambient particulate matter (PM) (Gordon et al. 1999; Sioutas et al. 1995), allowing toxicologic studies to be conducted at higher than ambient concentrations. This approach allows mechanistic characterization of PM effects and identification of causative constituents. One of the limitations of this approach, however, is that the physicochemical properties of CAP and ambient PM may differ because the overall enrichment of CAP may depend on the size of incoming particles.

A review of animal toxicologic studies involving CAP exposures has shown inconsistencies, and generally subtle health effects. Moreover, there is a lack of correlation between health end points and PM mass or its causative components (Clarke et al. 2000; Gordon et al. 2000; Kodavanti et al. 2000a; Saldiva et al. 2002; Smith et al. 2003). However, in clinical studies, CAP effects as determined by analysis of blood/plasma markers and constriction of arteries have been readily apparent (Ghio et al. 2003; Huang et al. 2003; Urch et al. 2004). These differences could be due to greater human sensitivity as well as reduced variability because the subject exposed to CAP most often serves as his or her own control for measured biologic variables. Repeated sampling is technically difficult with most end points in animals.

Although the animal studies using model particulate samples have provided links between specific constituents and biomarkers of health effects (Kodavanti et al. 1998), they often have been criticized for being irrelevant to human scenarios because of the exposure methods, dosages, or unrealistic composition of particles. CAP exposure studies, in contrast, are difficult to reproduce because of the varying dynamics of ambient PM concentration and composition. Therefore, without marked and reproducible biologic effects of ambient PM, it has been difficult to investigate mechanisms and causality. CAP studies performed using identical exposure protocols and novel biomarkers generally need to be evaluated for consistencies in concentration or composition-related trends.

On the basis of studies conducted using combustion and ambient particles, metals have been shown to comprise a class of significant causative constituents of PM health effects (Dye et al. 1999; Kodavanti et al. 1998). Metals have been ubiquitously detected in ambient PM, albeit at very low concentrations (Harrison and Yin 2000; Kodavanti et al. 2000a; Saldiva et al. 2002; Smith et al. 2003). The major metallic components of ambient PM at different locations include iron, silicon, aluminum, copper, and zinc. Recently, ambient organics have also been implicated in adverse health effects (Carvalho-Oliveira et al. 2005). The specific health effects of each of these components and their potential interactions in the environment and at the cellular level are not fully understood.

In this article, we describe multiple exposure studies using spontaneously hypertensive (SH) and Wistar Kyoto (WKY) rats. These studies were designed to identify consistency in the pattern of biologic response to correlate with CAP mass and composition, and potential susceptibility factors. We hypothesized that SH and WKY rats respond differentially to CAP and that selected biologic indicators reflect these differences in a strain-specific manner. We also explored whether CAP effects on biologic end points related better to mass or to putative causative constituents.

## Materials and Methods

### Animals.

Healthy, male, 10- to 12-week-old, normotensive WKY and SH (SHR/NCrlBR) rats (derived from WKY rats by segregation of the hypertensive trait and inbreeding) were purchased from Charles River Laboratories (Raleigh, NC). All rats were maintained in an isolated animal room in an animal facility approved by the Association for Assessment and Accreditation of Laboratory Animal Care [maintained at 21 ± 1°C, 50 ± 5% relative humidity (RH), 12-hr light/dark cycle] for 1–2 weeks of quarantine and nonexposure periods. All animals received standard (5001) Purina rat chow (Dyets, Inc., Bethlehem, PA) and water ad libitum except during CAP exposure periods of 4–5 hr. The U.S. Environmental Protection Agency (EPA) Animal Care and Use Committee approved the protocol for the use of rats in inhalation studies.

### CAP exposure.

Six repeat studies of 4 hr/day, 1-day exposures were conducted between 17 October and 16 November 2000, and seven repeat studies of 4 hr/day, 2-day exposures were conducted between 27 August and 24 October 2001. Rats in each study (SH rats for 1-day exposure studies, and SH and WKY rats for 2-day exposure studies) were computer randomized into two groups after sorting them from low weight to high weight to ensure similar means and distribution. One group was exposed to clean air, and the other to CAP using the U.S. EPA fine-mode CAP exposure system (1-day exposure studies; *n* = 5–9 rats per group, and 2-day exposure studies; *n* = 4–5 rats per group) (Sioutas et al. 1995). The series of four virtual impactors produced an empirical ambient fine-mode (≤ 2.5 μm) particle concentration enhancement of 40–60 times ambient levels in the exposure chamber. Animals were exposed generally between 0830 and 1330 hr for a total period of 4 hr during each exposure day [see Supplemental Material for details (http://ehp.niehs.nih.gov/docs/2005/7868/supp.pdf)].

Outside environmental conditions were continuously monitored using a weather station (Weather Monitor II; Davis Instruments, Haywood, CA) sited within 150 ft of the CAP system inlet. Ambient temperature, RH, dew point, wind speed, wind direction, and barometric pressure were recorded at 30-min intervals during each exposure. Control and exposure chamber temperature and RH were measured continuously (Omega RH-411 temperature and RH Thermo hygrometers; Omega Engineering, Stamford, CT).

A superimposed map of daily wind direction and speed for all 2001 exposure days was prepared from individual maps obtained from the World Wide Web–based Real-Time Environmental Applications and Display System (READY). This system has been developed for accessing and displaying meteorologic data on the National Oceanic and Atmospheric Administration (NOAA) Air Resources Laboratory web server (NOAA 2004).

### CAP organic and elemental analysis.

Samples for analysis of CAP mass concentration were collected on preweighed Teflon filters for the duration of each exposure. Postexposure, filters were weighed and concentrations determined by sample mass/sample flow volume (μg/m^3^). Ambient levels of total suspended particulate and fine-mode (≤ 2.5 μm) particles were measured gravimetrically using Teflon filters (2.0 μm, 37 mm, and 47 mm Teflo, R2PJ037, and R2PJ047; Pall Corp., East Hills, NY). These filters sequester particles > 0.3-μm in size with 99.7% efficiency [more details in Supplemental Material (http://ehp.niehs.nih.gov/docs/2005/7868/supp.pdf)].

Organic and elemental carbon contents of CAP collected on quartz filters were determined using National Institute of Occupational Health (NIOSH) thermal-optical method 5040 (Sunset Laboratory, Tigard, OR). To determine soluble ion and elemental content, we extracted each Teflon filter in distilled water for 1 hr and centrifuged the extracts at 17,000 × *g* for 30 min. We removed two aliquots of each supernatant and analyzed the first aliquot as is for sulfate and nitrate content using ion chromatography (McGee et al. 2003). We acidified the second aliquot to a pH < 2.0 using concentrated nitric acid to keep soluble metal salts in soluble form. We then analyzed the acidified extracts for elemental content using inductively coupled plasma–mass spectroscopy (ICP-MS) (McGee et al. 2003).

### Whole-body plethysmograph data acquisition and analysis.

We employed a barometric whole-body plethysmograph system (Buxco Electronics Inc., Sharon, CT) to obtain data on pulmonary ventilation for 2-day exposure studies. This methodology permitted monitoring of a number of ventilatory parameters, including breathing frequency (*f* ), tidal volume (Tv), minute ventilation (MV), peak expiratory flow (PEF), peak inspiratory flow (PIF), inspiratory time (Ti), expiratory time (Te), pause (PAU), and enhanced pause (PENH) (Tankersley et al. 1997). Unrestrained, free-moving animals were placed in individual chambers and allowed 1 min to settle down followed by 5 min of monitoring. We analyzed the data from each animal before the first CAP or air exposure and after the last exposure.

### Necropsy and sample collection.

Necropsies were performed within 3 hr after exposure in six 1-day exposure studies except for the fourth study where necropsies were performed 18–20 hr postexposure. All necropsies were performed 18–20 hr after the second exposure in 2-day exposure studies. At designated time points rats were weighed and anesthetized with sodium pentobarbital (50–100 mg/kg, intraperitoneally). Blood was collected from the abdominal aorta, directly into blood collection tubes containing EDTA (for complete blood counts) or citrate as anticoagulants (for plasma protein analysis). The trachea was cannulated and the left lung was tied. The bronchoalveolar lavage was performed as described previously (Kodavanti et al. 2000b).

### Blood chemistry and cytology.

Plasma fibrinogen and complete blood counts were performed by the University of North Carolina Memorial Hospital Core Facility (Chapel Hill). Each blood sample containing citrate anticoagulant was centrifuged at 4,500 rpm for 10 min at 4°C. Plasma was then analyzed for fibrinogen as described by Gardner et al. (2000). The complete blood counts were performed on a Technicon H-2 hematology analyzer (Bayer Corp., Tarrytown, NY) using Bayer Technicon reagents. Angiotensin-converting enzyme (ACE) activity was measured in citrated plasma using reagents and controls from Sigma Diagnostics (St. Louis, MO). C-Reactive protein (CRP) was measured in citrated plasma using an SPQ II kit that contained its own calibrations and controls (Diasorin Inc., Stillwater, MN). The ACE activity and CRP assays were adapted for use on the Cobas Fara II clinical analyzer (Hoffmann-La Roche, Branchburg, NJ).

### BALF analysis for determining lung injury.

We used one aliquot of whole bronchoalveolar lavage fluid (BALF) to determine total cell counts (Coulter counter; Coulter Inc., Miami, FL); a second aliquot was centrifuged (Shandon 3 Cytospin; Shandon, Pittsburgh, PA) to prepare cell differential slides. We dried the slides at room temperature and stained them with LeukoStat (Fisher Scientific Co., Pittsburgh, PA). Macrophages and neutrophils were counted using light microscopy (> 200 cells counted per slide). We centrifuged the remaining BALF at 1,500 × *g* to remove cells, and analyzed the supernatant fluid for markers of lung injury. Total protein was analyzed using Coomassie Plus Protein Assay Kit (Pierce, Rockford, IL). We analyzed BALF albumin using a commercially available kit (Diasorin). Lactate dehydrogenase (LDH) activity (U/L) was determined using Kit 228 from Sigma Chemical Co. (St. Louis, MO). We measured *N*-acetyl glucosaminidase (NAG) activity (U/L) using a kit and standards from Roche Diagnostics (Indianapolis, IN). γ -Glutamyltransferase (GGT) activity was measured using a kit from Thermo Trace Ltd. (Melbourne, Australia). These assays were adapted for use on the Cobas Fara II (Hoffmann-La Roche) clinical analyzer. We mixed an aliquot of BALF with an equal volume of 6% perchloric acid and vortexed. After standing on ice for 10 min, it was centrifuged (14,000 × *g*) for 10 min (4°C), and then supernatants stored at –80°C. We determined total glutathione and ascorbic acid as described previously (Kodavanti et al. 2000b).

### Determination of cytokines in BALF using enzyme-linked immunosorbent assay.

WKY rat BALF samples from all 2-day exposure studies were analyzed for interleukin-6 (IL-6), tumor necrosis factor-α, and macrophage inflammatory protein-2 using a sandwich enzyme-linked immunosorbent assay (ELISA) technique. Rat-specific cytokine assay kits were obtained from Biosource International (Camarillo, CA) and were used in performing ELISA. We ran each sample in triplicate. Sample optical density values were measured at 450 nm wavelength using microtiter plate reader (SpectraMax Pro 340PC; Molecular Devices, Sunnyvale, CA). This system uses SoftMax Pro (version 2.6.1; Molecular Devices) software to run the plate reader and analyze the data.

### Statistics.

For the data analysis of the 1-day exposure studies, we assumed a homogeneous variance for blood lymphocytes, platelets, hematocrit, and hemoglobin. Ranks were used to determine significance levels for all other biologic parameters where heterogeneity of variance was apparent. To determine significance levels, we used a crossed-design analysis of variance (ANOVA) with an interaction term.

Because of the small number of rats used per group in each of the 2-day exposure studies, the variance was heterogeneous, and therefore, we performed no statistical testing to determine study-to-study differences. We pooled the data into responses related to rat strain and exposure group and performed statistical analysis to determine if there were an interaction between exposure and seven studies. If there was no interaction, then we performed further ANOVA to determine if the strains responded differently for each variable. If there was no interaction, then we tested strain and exposure differences using ranks in ANOVA. Each strain was used separately in an ANOVA to determine significant differences between air and CAP exposures.

For the 2-day studies, we used averages of 2- to 4-hr exposures for mass and composition in all correlation analyses. We determined correlations for ambient air chemistry values using the Pearson correlation coefficient and also the Spearman method. Spearman correlations tend to reduce the influence of very large or very small measurements.

## Results

### Composition of exposure atmospheres.

The atmospheric conditions, before PM is concentrated, can influence its chemical composition during formation. All 1- and 2-day exposures occurred between 0830 and 1330 hr in an attempt to minimize this factor. The daytime high atmospheric temperatures were in 70–79°F except for the last exposure day (52°F), with the difference between daytime high and nighttime low temperatures being 17–28°F during 1-day exposure studies. In 2-day exposure studies, the daytime high temperatures reached 80–89°F, and the difference between daytime high and nighttime low temperatures was 11–30°F. Atmospheric humidity varied from 100% to < 50%. The mean temperature of control and CAP chambers varied within 2.5% and RH within 25% during all exposures.

### Elemental and organic composition of CAP.

Particle size range did not change significantly between 1- or 2-day exposure studies except for a slightly larger size during the 2-day exposure studies (Table 1). During 1-day exposure studies, generally high CAP concentrations were achieved with only a small variation between exposure days (Table 1, studies 1–6). This hindered the ability to establish relationships between components and mass, or within components. During seven 2-day exposure studies, low CAP concentrations were achieved except for the first study, and the exposure concentrations varied markedly between studies (Table 1, studies A–G).

The metal analysis included only water-soluble elements. Of many elements measured (aluminum, arsenic, barium, beryllium, cadmium, cobalt, copper, lead, manganese, nickel, silver, titanium, and zinc), the most abundant were aluminum, zinc, and copper (Table 1). Silica, sodium, and iron, which are also likely among the most abundantly detected metals, were not measured. The sulfate concentration was lowest when the lowest CAP concentrations were achieved (Table 1, 2-day exposure studies A–G). When the highest chamber concentration of CAP was achieved, the sulfate concentrations were predominant (Table 1, study A).

**Figure 1:**
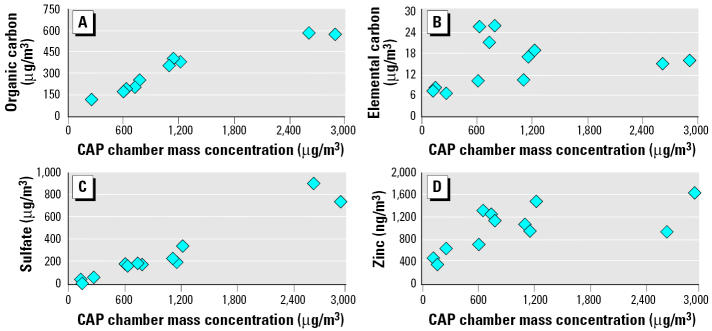
Correlations between CAP mass concentration in chambers and the levels of potential causative constituents (2-day exposure studies). The *x*-axis represents the mass values obtained during first and second day for each study (except that data for first day in study B are not available). The correlations with CAP mass are (*A*) organic carbon, *r* = 0.95, *p* = 0.0001; (*B*) elemental carbon, *r* = 0.18, *p* = 0.54; (*C*) sulfate, *r* = 0.94, *p* = 0.0001; (*D*) zinc, *r* = 0.64, *p* = 0.014.

The components that accounted for > 50% of CAP mass in all samples were sulfate, organic carbon, and elemental carbon (Table 1, 2-day exposure studies A–G). Organic carbon concentrations were 10–20 times higher than elemental carbon, considering samples from both years, and were significantly associated with CAP mass (Figure 1A). However, we observe no linearity between elemental carbon and CAP mass, as determined for the 2-day exposure studies (Figure 1B). The regression analysis of the samples from 2-day exposure studies indicated correlations between sulfate and CAP mass (Figure 1C). Significant correlations also existed between zinc and CAP mass (Figure 1D). Using CAP composition data of 2-day exposure studies, these four variables, organic carbon, elemental carbon, sulfate, and zinc, compared with mass concentrations produced correlations of 0.95, 0.18, 0.94, and 0.64, respectively (Figure 1). As expected, nitrate concentrations were low compared with sulfate. CAP nitrate concentrations varied between 37 μg/m^3^ (27 October 2000) and as low as 4 μg/m^3^ (2–3 October 2001). However, there appeared to be no linear relationship between nitrate levels and the mass (Table 1).

**Figure 2:**
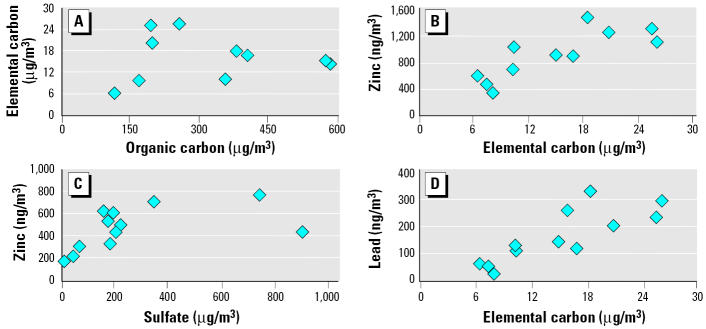
Interrelationships between concentrations of major constituents of CAP during 2-day exposure studies. A linear regression model was applied to determine if changes in the levels of major components were interrelated. The *x*- and *y*-axes depict values for different components during first and second day for each study (except that first-day data in study B are not analyzed). The correlations within components are (*A*) organic carbon versus elemental carbon, *r* = 0.02, *p* = 0.95; (*B*) elemental carbon versus zinc, *r* = 0.72, *p* = 0.006; (*C*) sulfate versus zinc, *r* = 0.55, *p* = 0.042; (*D*) elemental carbon versus lead, *r* = 0.82, *p* = 0.0006.

When individual CAP components were compared, it was apparent that organic and elemental carbon correlated poorly (*r* = 0.02) (Figure 2A). However, organic carbon mass correlated with sulfate and zinc but not with aluminum (data not shown). Zinc levels correlated well with elemental carbon (Figure 2B) and sulfate (Figure 2C), demonstrating correlations of 0.72 and 0.82. Correlations also existed between lead and elemental carbon (Figure 2D). When levels of metals in CAP were calculated per given CAP mass (microgram per milligram), it was apparent that aluminum, copper, and zinc were enriched severalfold on the PM during 2-day exposure studies B–G (Table 2). In these same studies organic carbon was also enriched to a smaller degree when expressed per milligram of CAP (Table 2).

**Figure 3:**
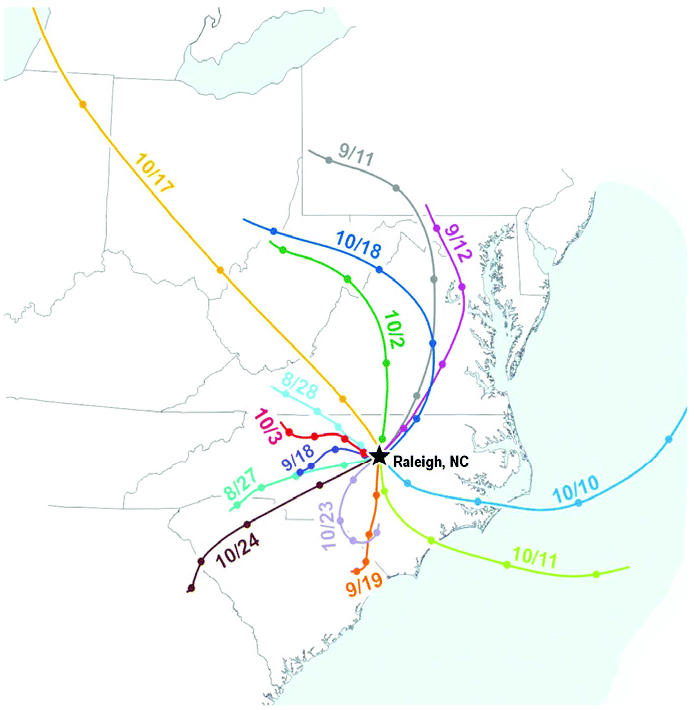
A map of superimposed weather trajectories for all 2-day CAP exposure studies during 2001. Individual backward weather trajectories were obtained from NOAA (2004). The lines pointing to Raleigh, North Carolina, show migration of air for 24 hr before 0900 hr on each exposure day. The distance between two color-matching markings on each line represents the distance traveled by air for 6 hr.

To determine the potential direction of ambient particle migration and their source, we evaluated backward weather trajectories (Figure 3). The wind direction during 10 and 11 October 2001 was from the east, primarily coming from Atlantic Ocean; during this time, there was the least amount of particles and sulfate concentrated in the chamber (Table 1, 2-day exposure studies A–G). There was no other noticeable consistency in wind pattern, speed, or direction that could be linked to specific particle composition and biologic effects.

### Baseline rat strain differences in biologic end points.

The mean values of all seven combined 2-day exposure studies for WKY and SH rats of control and CAP groups are given in Tables 3 and 4, and in Supplemental Material (http://ehp.niehs.nih.gov/docs/2005/7868/supp.pdf). SH and WKY rats differ markedly in several breathing parameters (Table 3). As we have shown previously (Kodavanti et al. 2000b, 2002), baseline values of several pulmonary markers also differ between WKY and SH rats. The levels of neutrophils, protein, and albumin were higher, but antioxidants such as glutathione, ascorbate, and uric acid were lower in BALF of SH rats than in WKY rats (Table 4). All the hematologic values assayed were higher in SH rats [Supplemental Material (http://ehp.niehs.nih.gov/docs/2005/7868/supp.pdf)], which is consistent with hypertensive disease (Bianchi et al. 1986).

### CAP-induced changes in breathing parameters.

**Figure 4:**
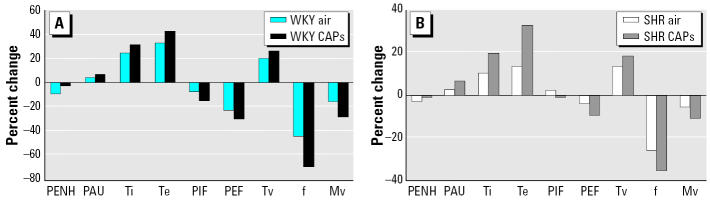
Effect of CAP exposure on breathing parameters in WKY (*A*) and SH (*B*) rats. Abbreviations: *f*, breathing frequency; Mv, minute volume; PAU, enhanced pause; PEF, peak expiratory flow; PENH, enhanced pause; PIF, peak inspiratory flow; Te, expiratory time; Ti, inspiratory time; TV, tidal volume. The *y*-axis represents percent change from preexposure values. Animal responses from all seven 2-day exposure studies were combined for WKY–air, WKY– CAP, SH–air, and SH– CAP groups, and percent change values represent mean of 28–35 animals/group.

Net body weight gains after CAP exposure did not change in any of the strains or exposure groups (data not shown). We measured breathing parameters for the 2-day exposure studies in each rat before and after CAP exposures to allow for paired analysis (Figure 4A,B). Although overall patterns of changes induced by CAP were consistent in six of seven studies, no individual study group reached significance with regard to changes in breathing parameters. The data for all air and all CAP groups were therefore combined for statistical analysis. Significant differences were noted in a number of breathing parameters by this maneuver (Table 3). The paired analysis included the percentage difference from baseline for all control and CAP groups for each rat strain. SH rats exposed to CAP demonstrated a significant overall increase in inspiratory time (Ti) and expiratory time (Te) relative to those of air controls (Figure 4B). These parameters were not significantly different in WKY rats (Figure 4A).

### Biochemical and inflammatory indicators of pulmonary injury and blood/plasma markers.

None of the biochemical and inflammatory parameters analyzed in the six 1-day exposure studies showed a consistent change. Five studies showed a marginal increase in plasma fibrinogen, which was not significant (data not shown).

**Figure 5:**
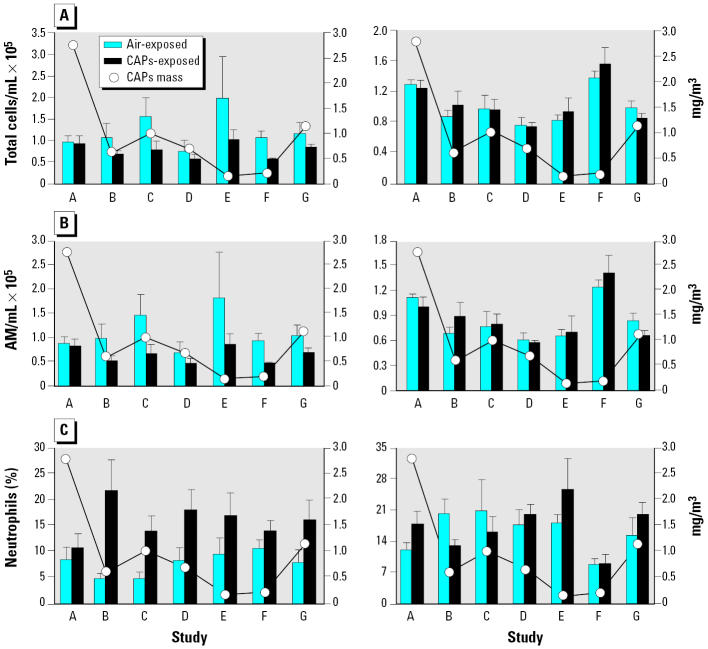
Inflammatory cells in BALF from WKY and SH rats after filtered air or CAP exposure in relation to mass concentrations of CAP. (*A*) Total cells. (*B*) Alveolar macrophages (AM). (*C*) Neutrophils. A–G on the *x*-axis denote individual 2-day exposure studies. The line graphs indicate mean mass concentrations of CAP in chambers during each study, with the response variable plotted on the right *y*-axis. Values represent mean ± SE of four to five animals per group during each study.

Although statistically not significant in each study, many parameters showed consistent CAP effects in a rat strain–specific manner during 2-day exposure studies. Combining data for all seven studies for those parameters demonstrated significant CAP effects [Table 4 and Supplemental Material (http://ehp.niehs.nih.gov/docs/2005/7868/supp.pdf)]. Total lavageable cells decreased in all studies except for the first one (study A) where the highest CAP concentration occurred (Figure 5A). This decrease in total cells was observed only in WKY rats and was associated with a decrease in lavageable macrophages (Figure 5B). The other effect we observed in WKY rats was the increase in neutrophils (Figure 5C) in all except for the first study when CAP concentrations were high. Thus, for the WKY rats, total cell count, alveolar macrophages, and neutrophils (%) were significantly different (*p* = 0.01, 0.0001, and 0.004, respectively).

SH but not WKY rats demonstrated a consistent increase in GGT activity in all studies except for the first one [Supplemental Material (http://ehp.niehs.nih.gov/docs/2005/7868/supp.pdf)]. GGT is a membrane-bound enzyme thought to indicate cell membrane integrity (Ernst et al. 2002). The plasma fibrinogen levels were higher in five of seven exposures in SH rats [Supplemental Material (http://ehp.niehs.nih.gov/docs/2005/7868/supp.pdf)], but surprisingly, during the first and the last exposure studies when the CAP concentrations were highest, the plasma fibrinogen levels did not increase. This effect was less consistent in WKY rats but was still statistically significant when all groups were combined.

**Figure 6:**
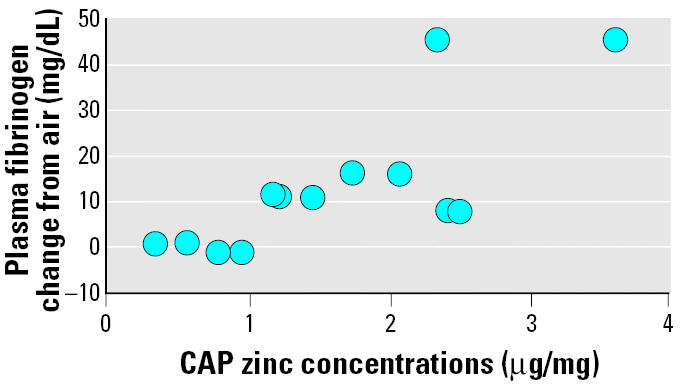
Correlation of zinc levels in the CAP (microgram per milligram) and CAP-induced plasma fibrinogen change over air control for 2-day exposure studies. Changes in plasma fibrinogen (CAP group – air group) were plotted against zinc levels of CAP as determined per milligram of mass during each day of exposure.

Biologic responses in WKY or SH rats were correlated with exposure variables such as CAP mass, organic and inorganic carbon, sulfate, and other major elemental constituents (microgram per cubic meter) using Spearman’s correlation. However, none of the variables showed significant correlation. Nevertheless, it should be noted that when biologic responses were correlated with metals such as aluminum, copper, and zinc normalized per unit mass of CAP (microgram per milligram), zinc correlated best with plasma fibrinogen in SH rats (*p* = 0.0023) (Figure 6). Correlations also existed between other metals and GGT levels but were less remarkable with neutrophils and total cell changes.

## Discussion

Because CAP composition is likely to vary significantly on different days during changing seasons, studies using identical protocols repeated over an extended time period provide an opportunity to identify modifying factors of biologic response. Here, we report that seven 2-day exposure studies (4 hr/day) conducted between 24 August and 27 October 2001, caused rat strain–specific and relatively consistent effects in a selected group of biologic variables. However, the changes in these variables were not correlated with CAP mass. The concentrations of aluminum, copper, and zinc were enriched severalfold per given mass of CAP (microgram per milligram) in studies that showed biologic response. Organic carbon was also enriched to a small degree in those studies but not sulfate, suggesting that physicochemical properties of CAP were different during those seven replicative studies. We hypothesize that CAP effects may be revealed only when unique CAP composition is formed (metal–organic enriched). However, direct mechanistic linking of biologic responses to a given constituent remains a challenge because most routinely used biologic variables in animals show small effects, and generally there is limited data availability.

There are potential explanations for the lack of effect of CAP on health end points in 1-day exposure studies. The exposures occurred only for 1 day (4 hr), and the responses were determined immediately after the exposure except in one replicate when necropsy was done 1 day postexposure. The higher and more variable baseline levels of neutrophils in SH rats may also have obscured any modest CAP effects, and the time to necropsy may have been too short to induce the migration of neutrophils in the lung.

Although neutrophilic inflammation and decreases in number of lavageable macrophages were apparent in WKY rats in most of 2-day exposure studies, no correlation existed with mass concentration. On the contrary, at the highest CAP levels no increase in neutrophils was apparent, suggesting that within a given range of concentrations, PM mass may not be the primary determinant of biologic response in the rat. The lack of correlations between mass and biologic end points has been noted in other studies (Kodavanti et al. 2000a; Saldiva et al. 2002; Smith et al. 2003). Potential interactions between constituents of CAP in the air or at the cellular level may significantly alter the toxicity of constituents.

Variation in CAP composition is governed by the atmospheric transformation, transport of emissions from downwind power and industrial plants, vehicular emissions, domestic combustion activities, forest fires, and a range of natural sources. In the 2-day exposure studies we observed a significant correlation between CAP mass and several of its components (expressed as microgram per cubic meter), such as sulfate, organic carbon, and zinc, but not other metals such as aluminum, copper, and lead. The significant correlation between elemental carbon and zinc points to the contribution by vehicular emissions. Also, the air shed in Research Triangle Park, North Carolina, is likely to have a significant sulfate contribution from regional transport over the nearby highway.

Given the significant differences in CAP mass and the health effects during 2001, we predicted that the weather trajectories would provide insight into the potential sources of CAP components. The composition of particles coming from the east (Atlantic Ocean) during 2 exposure days was different from that of particles migrating from the northwest and southwest. High sulfate content of CAP during southwest and northwest winds, and reduction thereof, during 2 days when wind was coming from the east, was potentially linked to contribution by industrial activities.

A significant discrepancy was noted in gravimetric measurement of CAP mass and total mass of components. This discrepancy could be due to inaccuracy in measurement of organic carbon as a mass of organic content. It has been suggested that the discrepancy in unaccountable PM mass stems, from the imprecision of organic carbon measurements, and from organic carbon concentration not representing total organic mass (Andrews et al. 2000). To obtain more accurate estimates of the most accountable CAP mass, Turpine et al. (2000) suggested that organic carbon concentrations be multiplied by a factor of 1.4. It is an estimate of the average molecular weight of organic mass per gram of organic carbon in atmospheric particle samples (Turpine et al. 2000). However, this multiplicative factor can vary depending on the source of ambient PM.

In search of CAP compositional factors that might have caused biologic effects, we noted that the water-soluble elements such as aluminum, copper and zinc, when expressed per milligram of CAP mass, were enriched severalfold in 2-day exposure studies when positive biologic responses were evident. The plasma fibrinogen changes in SH rats better correlated with the levels of metals (per mg CAP), especially water-soluble zinc. This relationship has also been recently demonstrated in humans exposed to CAP from the nearby town of Chapel Hill (Huang et al. 2003). The levels of organic carbon per milligram of CAP also were slightly enriched and were correlated with changes in fibrinogen. These data suggest that the overall physicochemical makeup rather than particle mass may be important, especially considering water-soluble metallic constituents and organics. We can hypothesize that metal and organic-enriched PM may cause greater tissue damage than particles having low overall metal–organic mass because concentration of these causative components may reach high at the site of their deposition in the microenvironment of airways.

Because other CAP studies in the literature measured total metals using X-ray fluorescence (Saldiva et al. 2002; Smith et al. 2003), it is not possible to compare levels of major elemental CAP components such as aluminum, iron, silica, copper, and zinc because our studies focused on the water-soluble elements using ICP-MS (Kodavanti et al. 2000a). We can hypothesize that readily water-soluble metals induce acute effects, whereas less soluble metals on PM collected within phagolysosomes can slowly leach off and produce long-term health effects. Thus, our correlations of water-soluble components with health effects possibly reflect an acute and direct action of metals. However, the role of organics cannot be excluded because organics constitute a bigger portion of CAP mass relative to elements, and levels of organics were correlated with biologic end points. Determination of individual organic species is critical in identifying their roles in health effects. Also, because sulfate is one more major CAP constituent, its interaction with metals and organics and overall effect on physicochemistry can play a crucial role in biologic activity of particles.

Small but consistent CAP-related changes in various breathing parameters were noted across all seven studies in SH rats; however, these changes were not reflected as an enhanced pause (PENH) increase, believed to be associated with altered airway function. It is not clear whether the strain differences in CAP response are due to differences in the basal values for breathing parameters in these two strains. It is important to note that changes in breathing parameters in SH rats were not associated with CAP-induced neutrophilic inflammation because these later changes were not consistent in SH rats. It remains to be investigated whether the increases in inspiratory and expiratory times were related to CAP-induced autonomic stimulation.

The purpose of measuring a variety of biologic end points was partly to ascertain which biomarker best reflects fluctuations in CAP mass and components. We had hypothesized that SH rats would demonstrate specific susceptibility to the cardiovascular effects, whereas WKY rats would demonstrate a more apparent neutrophilic response because of their consistent low baseline values compared with those of SH rats (Kodavanti et al. 2002). As hypothesized, WKY but not SH rats demonstrated a clear difference in neutrophilic inflammation between air and CAP groups. Also, the increase in neutrophils in WKY rats was associated with a marked and consistent decrease in total lavageable macrophages. A possible explanation for this difference is that macrophages in WKY rats might have been activated after CAP exposure and thus difficult to remove by lavage. The alveolar macrophages in SH rats, however, may reside in an activated state because of underlying disease and thus cannot be further activated by CAP. Macrophages and the lung epithelial cells in SH rats more readily express Toll-like receptors than WKY rats (Gilmour et al. 2004).

The membrane-bound enzyme GGT is involved in transport of amino acids across cell membranes and has been used as an early marker of precancerous lesions and of glutathione synthesis activity (Ernst et al. 2002). The detection of this enzyme in BALF might indicate loss of cell membrane integrity (Ernst et al. 2002). GGT levels increased only in SH rats in 2-day exposure studies. However, LDH and NAG, enzyme indicators of cytotoxicity, were not increased in SH rats.

These multiple CAP studies conducted over 2 years have provided some insight into the role of CAP composition in relation to biologic effects and the sensitivity of two rat strains. It is clear that CAP mass concentrations were not driving the response in our experimental settings; rather, it appeared that the water-soluble metals and organic enrichment of particles might be more critical in eliciting acute health effects, especially the association of plasma fibrinogen increase with zinc and organic carbon. The WKY rat may be an appropriate animal model for CAP effects on inflammation, whereas SH rats may represent a better model to study systemic effects of CAP.

## Supplementary Material

Supplemental Material

## Figures and Tables

**Table 1:** Chamber CAP concentrations and its organic and leachable elemental composition during 1- and 2-day exposure studies.

Study	CAP exposure date	Chamber concentration (μg/m^3^)	Particle size (μm; mean ± SD)^a^	Sulfate (μg/m^3^)	EC (μg/m^3^)	OC (μg/m^3^)	NO_3_ (μg/m^3^)	Al (ng/m^3^)	Ba (ng/m^3^)	Cu (ng/m^3^)	Pb (ng/m^3^)	Mn (ng/m^3^)	Ni (ng/m^3^)	Zn (ng/m^3^)
1	17 Oct 2000	1,765	1.09 ± 1.31	583	22.6	359	29.6	1,578	229	948	300	153	45	1,398
2	18 Oct 2000	1,748	1.12 ± 1.28	883	16.9	306	10.1	711	154	236	181	85	26	683
3	20 Oct 2000	1,504	1.07 ± 1.35	463	19.0	288	7.8	1,383	628	5,045^b^	243	229	22	1,306
4	25 Oct 2000	1,138	1.16 ± 1.40	235	17.5	278	22.2	2,728	456	781	190	154	118	860
5	27 Oct 2000	1,388	1.19 ± 1.36	363	15.0	284	37.2	2,782	349	290	286	164	36	780
6	16 Nov 2000	1,176	1.09 ± 1.40	132	32.8	419	10.6	2,767	599	367	229	537	41	1,852
A	27–28 Aug 2001	2,758	1.27 ± 1.42	823	15.5	581	15.0	1,082	208	386	207	154	32.3	1,259
B^c^	11–12 Sep 2001	604	1.44 ± 1.48	188	10.3	169	5.7	699	212	514	134	118	7.9	704
C	18–19 Sep 2001	994	1.36 ± 1.50	264	22.2	320	7.4	1,463	324	573	318	161	38.5	1,295
D	2–3 Oct 2001	685	1.37 ± 1.49	181	23.2	225	4.0	1,542	351	846	224	188	13.3	1,283
E	10–11 Oct 2001	144	1.48 ± 1.42	10	8.1	ND	9.7	896	143	604	24	87	8.0	351
F	17–18 Oct 2001	199	1.39 ± 1.45	58	7.0	116	4.6	1,361	184	586	59	96	18.7	545
G	23–24 Oct 2001	1,129	1.44 ± 1.37	215	13.7	382	12.6	1,383	379	530	116	177	33.0	969

Abbreviations: EC, elemental carbon; ND, not determined; OC, organic carbon. Each study is listed in the order that it was performed (1–6 for 1-day exposure studies and A–G for 2-day exposure studies).

a Particle size represents aerodynamic diameter based on number count per cubic centimeter measured as dN/dlogDp (mean diameter weighted by number/logarythmic interval of particle diameter). A minimum of one sample was collected per hour of exposure. Particle size data were averaged first over the course of each exposure for 1-day exposure studies, and then over 2 days for 2-day exposure studies, except for the exposures of 11 September where only 1 data point existed.

b Note that this value for copper obtained was very high for no explainable technical reason.

c Data collected on 11 September are for exposures < 1 hr.

**Table 2:** Levels of organic and major elemental constituents per given mass of CAP during 1-and 2-day exposure studies (microgram/milligram).

Study/day^a^	Exposure year	OC	EC	Sulfate	Al	Cu	Pb	Zn
A	2000	203	12.8	330	2.7	0.5	0.17	0.8
B	2000	175	9.7	505	0.8	0.1	0.1	0.4
C	2000	191	12.6	308	3.0	NA	0.16	0.9
D	2000	244	15.4	207	11.6	0.7	0.17	0.8
E	2000	205	10.8	262	7.7	0.2	0.21	0.6
F	2000	356	27.9	112	21.0	0.3	0.19	1.6
A1	2001	199	5.5	256	1.3	0.1	0.09	0.6
A2	2001	244	5.8	345	1.4	0.2	0.06	0.3
B2	2001	279	17.0	311	3.7	0.9	0.22	1.2
C1	2001	334	33.6	232	8.4	0.7	0.39	1.4
C2	2001	315	15.2	287	4.1	0.5	0.28	1.2
D1	2001	308	40.0	255	10.1	0.9	0.38	2.1
D2	2001	273	28.5	273	4.7	1.5	0.28	1.7
E1	2001	NA	NA	57	111.5	4.8	0.17	2.4
E2	2001	NA	58.0	89	68.5	3.5	0.17	2.5
F1	2001	NA	56.1	373	24.8	3.8	0.41	3.6
F2	2001	434	24.3	247	22.8	2.5	0.24	2.3
G1	2001	351	14.6	179	10.0	0.7	0.11	0.8
G2	2001	323	9.4	203	3.2	0.2	0.1	0.9

Abbreviations: EC, elemental carbon; NA, not analyzed; OC, organic carbon.

a Numerals 1 and 2 refer to the first and second day of exposure, respectively. Note that during B2–F2 exposure days, levels of aluminum, copper, and zinc per given mass increased severalfold when compared with other exposure days.

**Table 3:** Combined mean values (± SE) obtained for breathing parameters in SH and WKY rats after exposure to filtered air or CAP for all 2-day exposure studies.

Test	Expression unit	WKY/air	WKY/CAP	SH/air	SH/CAP
Frequency (*f* )	Breaths/min	266 ± 8	239 ± 11	290 ± 11	279 ± 8**
Tidal volume (Tv)	mL	1.20 ± 0.04	1.30 ± 0.04	1.40 ± 0.05*	1.46 ± 0.06*
Minute volume (Mv)	mL/min	265 ± 8	247 ± 11	341 ± 10*	342 ± 10*
Peak expiratory flow (PEF)	mL/sec	12.2 ± 0.4	12.0 ± 0.5	16.9 ± 0.5*	17.5 ± 0.7*
Peak inspiratory flow (PIF)	mL/sec	16.6 ± 0.4	16.1 ± 0.6	22.7 ± 1.0*	22.6 ± 1.5*
Expiratory time (Te)	Sec	0.19 ± 0.01	0.24 ± 0.02	0.20 ± 0.02	0.21 ± 0.02
Inspiratory time (Ti)	Sec	0.12 ± 0.00	0.13 ± 0.01	0.10 ± 0.00	0.11 ± 0.00
Pause (PAU)	Nondimensional	0.62 ± 0.01	0.65 ± 0.02	0.64 ± 0.03	0.68 ± 0.03
Enhanced pause (PENH)	Nondimensional	0.45 ± 0.01	0.50 ± 0.02	0.50 ± 0.03	0.54 ± 0.03

These data represent the absolute value of breathing parameters obtained after air or CAP exposure. Because the data in this table do not compare the net difference between baseline values obtained before and after air/CAP exposure, CAP-related differences may not be as apparent as shown in Figure 4. The number of observations/rats for WKY filtered air or CAP groups were 28, and for SH, 35.

* Significant strain effect at *p* ≤ 0.05.

** Significant CAP effect at *p* ≤ 0.05.

**Table 4:** Combined mean values (± SE) obtained for pulmonary injury/inflammatory markers analyzed in BALF of WKY and SH rats after exposure to filtered air or CAP for all 2-day exposure studies.

Parameter	Expression unit	WKY/FA	WKY/CAP	SH/FA	SH/CAP
Total protein	μg/mL	184 ± 34	148 ± 8	285 ± 19*	291 ± 17*
Albumin	μg/mL	27.0 ± 7.8	19.9 ± 1.8	54.0 ± 4.3	54.1 ± 4.2
LDH activity	U/L	32.8 ± 1.6	29.9 ± 2.1	18.1 ± 1.6*	16.8 ± 0.5*
GGT activity	U/L	2.68 ± 0.21	2.92 ± 0.22	2.64 ± 0.15	3.55 ± 0.15**
NAG activity	U/L	3.20 ± 0.20	3.10 ± 0.20	2.24 ± 0.11*	2.54 ± 0.11*
Glutathione^a^	ng/mL	0.80 ± 0.09	1.11 ± 0.10	0.59 ± 0.04	0.65 ± 0.04
Ascorbate	ng/mL	738 ± 45	818 ± 51	373 ± 17*	382 ± 18*
Uric acid	ng/mL	72.3 ± 8.4	93.6 ± 9.1	38.2 ± 5.1*	43.4 ± 5.9*
Total cells	Cells/mL × 10^5^	1.22 ± 0.17	0.77 ± 0.06**	1.01 ± 0.05	1.04 ± 0.07
Macrophages	Cells/mL × 10^5^	1.12 ± 0.12	0.65 ± 0.06**	0.85 ± 0.05	0.87 ± 0.07
Neutrophils	Cells/mL × 10^5^	0.08 ± 0.01	0.11 ± 0.01**	0.15 ± 0.01*	0.17 ± 0.01*
IL-6^b^	pg/mL	17.2 ± 2.1	19.8 ± 1.4	ND	ND
TNF-α	pg/mL	11.2 ± 1.2	14.0 ± 1.3	ND	ND
MIP-2	pg/mL	354 ± 16	371 ± 16	ND	ND

Abbreviations: FA, filtered air; MIP-2, macrophage inflammatory protein-2; ND, not determined; TNF-α, tumor necrosis factor-α. The number of observations/rats for WKY filtered air or CAP groups were 28, and for SH, 35.

a Note that the levels of total glutathione are near the lower detectable levels and therefore variable.

b Also IL-6 was below the detection limit in BALF (detection limits: IL-6, 62.5 pg/mL; MIP-2, 80 pg/mL; TNF-α, 9.37 pg/mL).

* Significant strain effect at *p* ≤ 0.05.

** Significant CAP effect at *p* ≤ 0.05.
