# Supplementary material for: Consistent Pulmonary and Systemic Responses from Inhalation of Fine Concentrated Ambient Particles: Roles of Rat Strains Used and Physicochemical Properties
Source: Environ Health Perspect. 2005 Jun 27;113(11):1561–8. doi: 10.1289/ehp.7868 (PMC1310919; doi:10.1289/ehp.7868)
Supplement: Supplemental Material [file ehp0113-001561s1.pdf]

Supplemental material:

MS # 7868, UP Kodavanti, MC Schladweiler, AD Ledbetter, JK McGee, L Walsh, PS Gilmour, JW Highfill, D Davies, KE Pinkerton, JH Richards, K Crissman, D Andrews, and DL Costa

Consistent Pulmonary and Systemic Responses from Inhalation of Fine Concentrated Ambient Particles: Roles of Rat Strains Used and Physicochemical Properties

CAP Exposure Methods and Mass Analysis:

The concentrator exposure system utilizes virtual impactor technology in which entering air flows (~4000L/min) through a series of slit virtual impactors where minor flow concentrates ambient particles of ~0.1 to 2.5 µm (Sioutas et al., 1995). The virtual impactors were operated at approximately 4:1 major to minor flow ratio resulting in a concentration factor (including slit loses) of about 3 X for each stage. The series of 4 virtual impactors produced an empirical ambient PM2.5 concentration enhancement of 40-60 times ambient levels in the exposure chamber. Air containing real time CAPs was directed through an 80 L, Hinners style, stainless steel and glass exposure chamber with 7.5 air changes/hour. This CAPs chamber allowed a maximum of 9 rats in individual wire mesh cages. Chamber temperature and relative humidity were recorded periodically during the exposure.

Operation of the CAPs system produced a nominal negative pressure in the exposure chamber of approximately 12-14 inch H_2O. Clean air control animal exposures were conducted simultaneously in a similar chamber and under similar conditions. Air entering the control chamber was filtered to remove particles. Animals were exposed generally between 8:30 am and 1:30 pm for a total period of 4 hours during each exposure day. The animals were placed in a single layer of individual

stainless steel wire mesh cages sized per AAALAC guidelines. No food or water was provided during exposures. All animals were weighed before and after the exposure. Because the concentrator system can't be effectively operated during unfavorable weather condition (i.e., rain), all exposures were conducted during non-rainy days.

CAPs system inlet and chamber concentrations were also measured using filters (2.0 μm, 47 mm Teflo, for gravimetric mass concentrations; 47 mm pre-fired quartz for OC/EC; Sunset Laboratory, Tigard, OR), and a tapered element oscillating microbalance (TEOM model 1400a, Rupprecht & Patashnick Co., Albany NY), as well as laser photometer (DustTrak Aerosol Monitor model 8520, TSI, Inc., St. Paul, Mn). Inlet particle size was monitored using cascade impactor technology (MOUDI, model 110, MSP Corp., Shoreview, MN). Chamber particle size was measured using a TOF photometric monitor (Aerosizer Mach 2, Amherst Process Instruments, Hadley, MA). A minimum of 1 sample was collected per hour of exposure. Particle size data were averaged over the course of each exposure, and in case of 2001 studies over 2 days of 4 h each exposure. All filter sample volumes were measured by positive displacement using volumetric dry gas meters (Rockwell model S-110, Environmental Supply Co. Durham, NC). Mass sample filters were environmentally stabilized at 72 +/- 2° F, 30 +/- 10 % RH for a minimum of 24 hours prior to pre- and post-sampling weight measurements on a micro-balance (model C-33, Cahn, Beverly, MA).

Table: Combined mean values obtained for hematological parameters and serum proteins in WKY and SH rats following exposure to filtered air or CAPs for all 2-day exposure studies.

Parameter	Unit of expression	WKY/FA	WKY/CAPs	SH/FA	SH/CAPs
RBC	cells/mlx10^9	8.6±0.10	8.6±0.04	9.16±0.04	9.22±0.05
Hemoglobin	g/dl	14.7±0.10	14.7±0.10	14.7±0.1	14.8±0.1
Hematocrit	%	42.8±0.30	42.8±0.30	43.5±0.2	43.8±0.2
Platelets	cells/mlx10^6	632±14	636±6	971±12*	990±12*
Total WBC	cells/mlx10^6	2.82±0.12	2.64±0.11	3.06±0.14	3.11±0.13
Neutrophils[a]	cells/mlx10^6	0.21±0.02	0.18±0.02	0.23±0.02	0.28±0.02*
Lympocytes[a]	cells/mlx10^6	1.84±0.08	1.75±0.08	1.79±0.10	1.68±0.11
Monocytes[a]	cells/mlx10^6	0.58±0.04	0.54±0.03	0.84±0.05	0.93±0.06
Fibrinogen	cg/dl	208±3.0	216±2.0	232±3.0*	245±5*, **
CRP	μg/ml	38.1±0.4	38.3±0.4	37.3±0.3	37.7±0.3
ACE activity	units/liter (U/L)	84.7±1.9	81.7±0.8	93.2±0.7*	91.5±0.7*

All blood complete blood count parameters were measured using human clinical analyzer. RBC=red blood cells; CRP=C-reactive protein; ACE=angiotensin converting enzyme. The number of observations/rats for WKY filtered air (FA) or CAPs groups were 28, and for SH, 35. Values represent mean ± standard error. *Significant strain effect at P ≤ 0.05. ** Significant CAPs effect at P ≤ 0.05. [a]Note that total WBC count and values for neutrophils, lymphocytes and monocytes were slightly lower than what is reported in the literature (Kumar et al. 2000). This may be due to the instrumental settings, which might differ slightly for the rat on a hematology analyzer.

Figure: Differential effects of CAPs on plasma fibrinogen and bronchoalveolar lavage fluid (BALF) γ-glutamyl transferase (GGT) activity in WKY and SH rat.

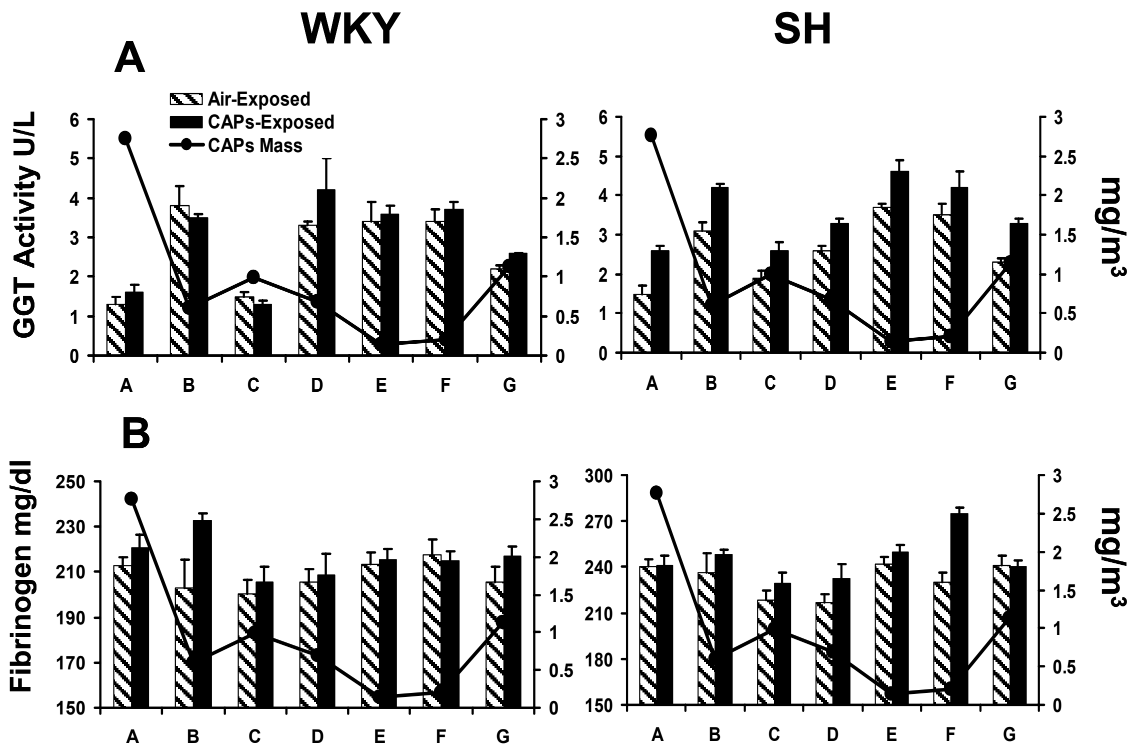

A-G on x-axis denotes the number given to individual 2-day exposure studies in chronological order. The line-graph indicates mass concentration of CAPs in chamber during each study with the response variable plotted on right Y-axis. Values represent mean ± SE of 4-5 animals/group during each study. The legend shown in A-WKY is also applicable to other graphs.

References:

Sioutas C, Koutrakis P, Burton RM. 1995. A technique to expose animals to concentrated fine ambient aerosols. Environ Health Perspect 103:172-177.

Kumar N, Savage T, DeJesus W, Tsong YY, Didolakar A, Sundaram K. 2000. Chronic toxicity and reversibility of antifertility effect of immunization against gonadotrophin-releasing hormone in male rats and rabbits. Toxicol Sci 53:52.
